# Patient reported outcomes after navigated minimally invasive hybrid lumbar interbody fusion (nMIS-HLIF) using cortical bone trajectory screws

**DOI:** 10.1097/MD.0000000000031955

**Published:** 2022-12-16

**Authors:** Kerim Hakan Sitoci-Ficici, Hongzen Jiang, Agrin Esmael, Daniel Ruess, Clemens Reinshagen, Uta Brautferger, Gabriele Schackert, Marek Molcanyi, Thomas Pinzer, Radovan Hudak, Jozef Zivcak, Bernhard Rieger

**Affiliations:** a Department of Neurosurgery, Dresden University Hospital, Dresden, Germany; b University Comprehensive Spine Center, Dresden University Hospital, Dresden, Germany; c Department of Neurosurgery, Chinese PLA General Hospital, Beijing, China; d Department of Neurosurgery, Cologne University Hospital, Cologne, Germany; e Department of Stereotactic and Functional Neurosurgery, Cologne University Hospital, Cologne, Germany; f Department of Neurosurgery, Brigham and Women’s Hospital, Harvard Medical School, Boston, MA; g Department of Urology, Rostock University Hospital, Rostock, Germany; h Institute of Neurophysiology, Medical Faculty, University of Cologne, Cologne, Germany; i Department of Neurosurgery, Medical University of Graz, Graz, Austria; j Department of Biomedical Engineering, Technical University of Košice, Koišce-Sever, Slovakia; k AMEOS Klinikum Halberstadt, Halberstadt, Germany.

**Keywords:** biokinemetrie, cortical bone trajectory, Lumbar interbody fusion, minimally invasive surgery, MIS-HLIF, PLIF, PRO, TLIF

## Abstract

Prospective observational study. To evaluate patient-reported outcomes after navigation-guided minimally invasive hybrid lumbar interbody fusion (nMIS-HLIF) for decompression and fusion in degenerative spondylolisthesis (Meyerding grade I-II). Posterior lumbar interbody fusion (PLIF) and transforaminal lumbar interbody fusion (TLIF) are well-known standard procedures for lumbar spinal fusion. nMIS-HLIF is a navigation-guided combined percutaneous and open procedure that combines the advantages of PLIF and TLIF procedures for the preparation of a single-port endoscopic approach. 33 patients underwent nMIS-HLIF. Core outcome measure index (COMI), oswestry disability index (ODI), numeric rating scale (NRS) back, NRS leg, and short form health-36 (SF-36) were collected preoperatively and at follow-up of 6 weeks, 3 months, 6 months, and 1 year. The impact of body mass index (BMI) was also analyzed. Computed tomography reconstruction was used to assess realignment and verify fused facet joints and vertebral bodies at the 1-year follow-up. 28 (85%) completed the 1-year follow-up. The median BMI was 27.6 kg/m^2^, age 69 yrs. The mean reduction in listhesis was 8.4% (*P* < .01). BMI was negatively correlated with listhesis reduction (*P* = .032). The improvements in the NRS back, NRS leg, ODI, and COMI scores were significant at all times (*P* < .001—*P* < .01). The SF-36 parameters of bodily pain, physical functioning, physical component summary, role functioning/physical functioning, and social functioning improved (*P* < .003). The complication rate was 15.2% (n = 5), with durotomy (n = 3) being the most frequent. To reduce the complication rate and allow transitioning to a fully endoscopic approach, expandable devices have been developed. The outcomes of nMIS-HLIF are comparable to the current standard open and minimally invasive techniques. A high BMI hinders this reduction. The nMIS-HLIF procedure is appropriate for learning minimally invasive dorsal lumbar stabilization. The presented modifications will enable single-port endoscopic lumbar stabilization in the future.

## 1. Introduction

Degenerative spondylolisthesis is a disease of elderly people.^[1]^ To cope with the increasing number of elderly patients undergoing lumbar stabilization, simple, easy-to-learn, safe, and effective minimally invasive surgical techniques are required. A single-port endoscopically assisted lumbar stabilization procedure is desirable. To enable a single-port endoscopic procedure and to ensure its acceptance in the field, existing surgical stabilization procedures must be modified step-by-step and supplemented by novel technical features and devices.

Spine surgeons are familiar with common standard open procedures, posterior lumbar interbody fusion (PLIF), and transforaminal lumbar interbody fusion (TLIF). However, open surgical techniques carry the risk of major muscle trauma, which has a negative impact on the outcome.^[2–7]^ Postoperative fibrotic changes in the paraspinal muscles play a key role in the development of postoperative back pain.^[8]^ Minimally invasive stabilization techniques reduce muscle trauma and protect muscle innervation through percutaneous implantation of pedicle screws. However, percutaneous pedicle screw insertions carry a greater risk of screw malpositioning and injury to neural structures. Minimally invasive procedures are associated with increased radiation exposure of the surgical team.^[9–12]^

Navigated minimally invasive (MIS) hybrid lumbar interbody fusion (nMIS-HLIF), a technique aimed at being the 1^st^ step toward a single-port endoscopic stabilization procedure, was developed in 2010 and combined benefits from PLIF and TLIF in 1 surgical procedure. nMIS-HLIF effectively enabled minimally invasive bilateral decompression (PLIF) and listhesis reduction via a single-sided surgical approach. To minimize the risks of increased radiation exposure and nerve tissue trauma from percutaneous insertion of screws, navigated MIS-HLIF was introduced and later supplemented by preoperative simulation.^[13,14]^

Despite the enormous technical progress in the last decade as well as the stable and good clinical results with minimally invasive procedures, there is still a certain reluctance in the field to abandon standard open surgery and to transition fully to minimally invasive procedures for treating degenerative listhesis. To address these doubts and to accelerate the development of a single-port endoscopic procedure for lumbar stabilization, we examined the results of the 1^st^ 33 nMIS-HLIF procedures performed by a neurosurgeon with little experience in the field of lumbar spine stabilization. This prospective observational study evaluated the radiological and clinical outcomes of nMIS-HLIF in patients up to 1 year after surgery. In addition to functional measures and complications, the effects of body mass index (BMI) on listhesis reduction and postoperative quality of life were evaluated. Experienced challenges and complications served as a guide for developing single-port lumbar endoscopic stabilization, and this study further introduces our group´s newest technical developments to facilitate this development.

## 2. Methods

### 2.1. Ethical statement

This prospective observational study was approved by the local ethics committee and conducted in accordance with the Declaration of Helsinki.

### 2.2. Surgical technique of nMIS-HLIF

**Figure 1.**
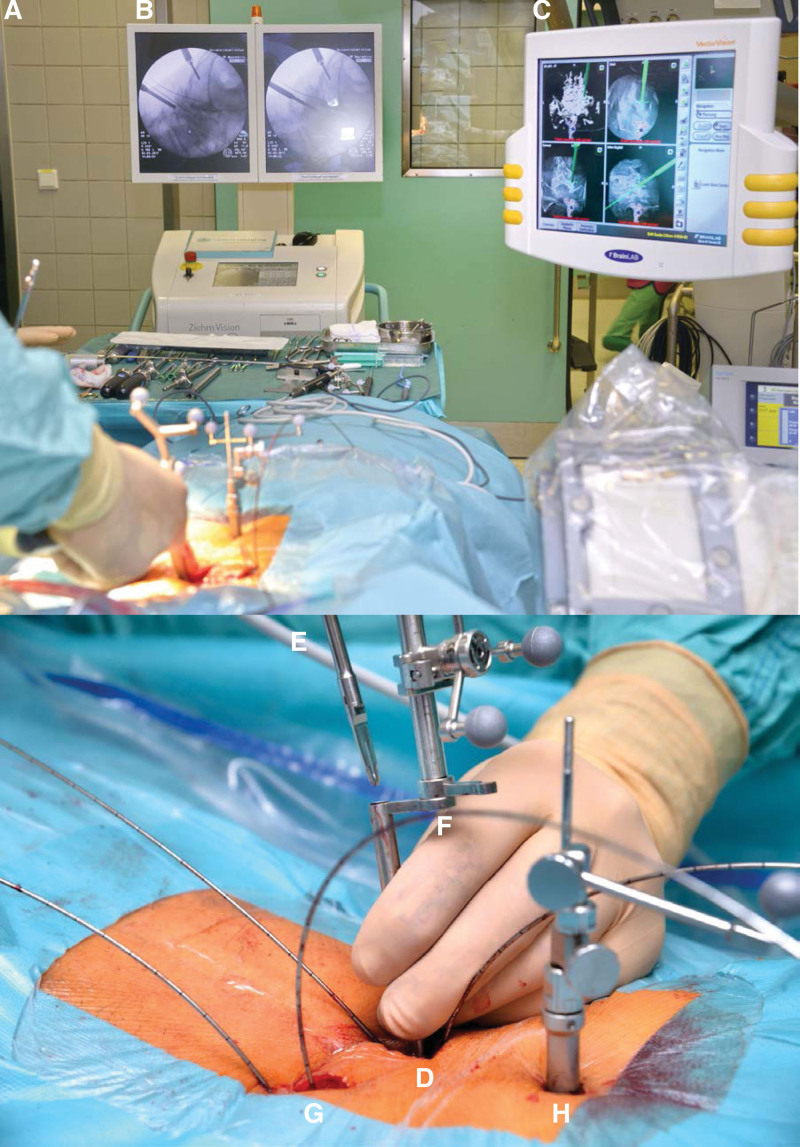
1A–1H. Intraoperative images showing the surgical setting (A) with radiologically guided (B) and navigation-assisted (C) insertion of K-wires. A 3 to 4 cm median incision (D) is sufficient for the laminotomy and ipsilateral and contralateral decompression in crossover technique. In this case, the decompression is done with the help of a navigated ultrasonic knife (E). Through the navigated sleeve (F) fits a high speed drill just as well. Navigated endoscopy armed with ultrasonic knife and high speed drill is another technical feature towards single port endoscopically assisted lumbar fusion because it enables effective endoscopic segmental decompression in an appropriate surgical time. Contralaterally, 2 incisions (G) of approximately 5 to 10 mm are made at a distance of 3 cm from the midline to perform percutaneous pedicle screw placement. Navigation array in situ (H).

**Figure 2.**
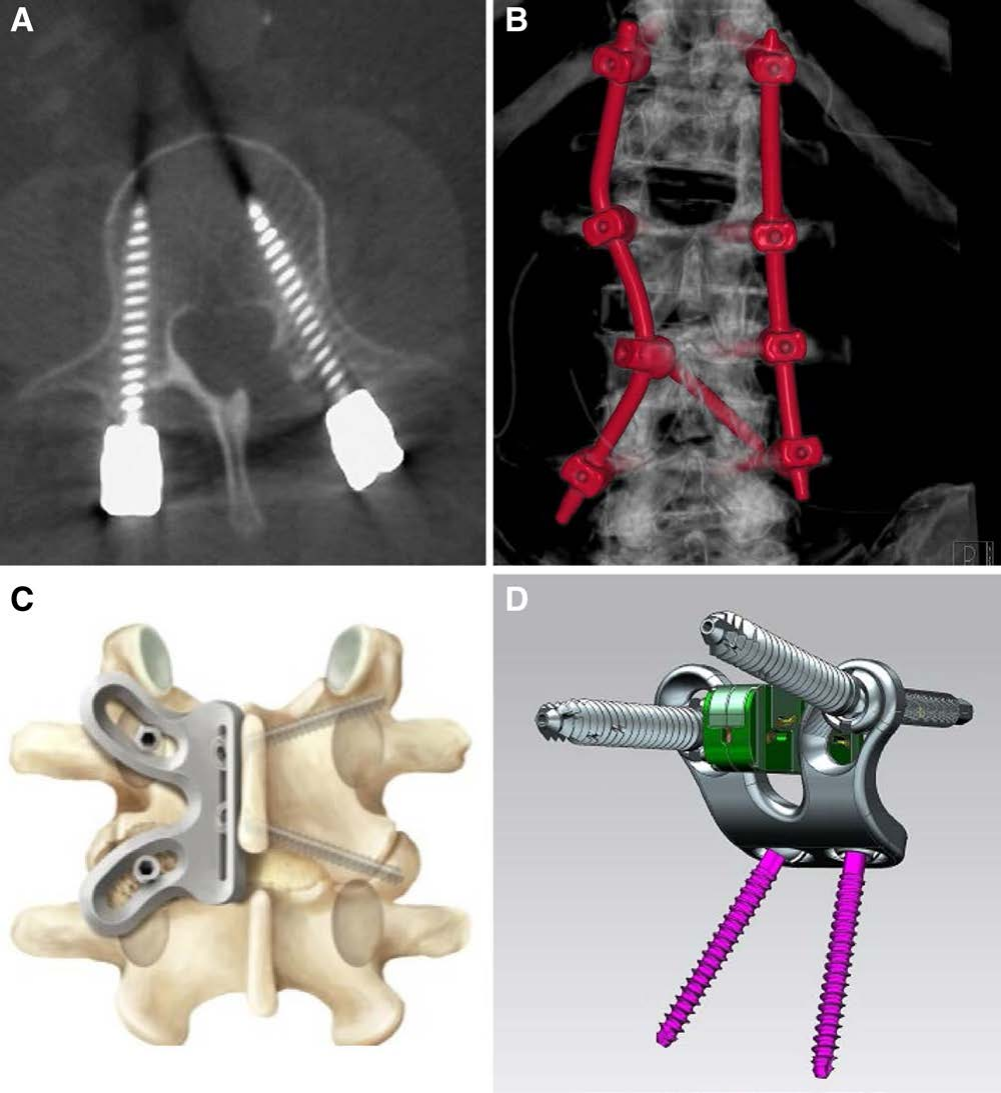
(A) The combination of a more straightforward cortical bone trajectory screw (right) and a common dorsolateral pedicle screw (left) was the first surgical modification in nMIS-HLIF to enable a smaller approach. (B) A combination of more straightforward cortical bone trajectory screws and 1 transfacet screw in a patient with pedicle metastases for a better biomechanical behavior of the stabilization system is shown. (C) The combination of straightforward cortical bone trajectory screws (right) with an upper laminar and a lower transfacet screw (left) resulted in the open MIS-VLIF stabilization system. This open MIS-VLIF can further be combined with a patented specially designed plate (K-plate) which facilitates screw-insertion and repositioning as well as expandable devices implantation as shown in (D) thereby serving as an intermediate technical step towards the endoscopic single port lumbar fusion in degenerative listhesis (expandable cage is shown in green). nMIS-HLIF = navigated minimally invasive hybrid lumbar interbody fusion, MIS-VLIF: minimally invasive vector lumbar interbody fusion.

**Figure 3.**
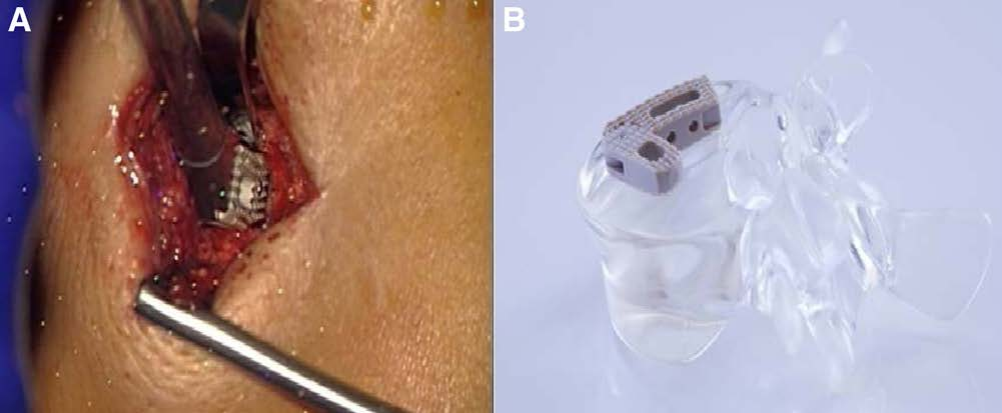
(A) A specially for MIS-HLIF in 2010 designed polyether ether ketone (PEEK) non-expandable cage (Synchro; ARCA-MEDICA, Neuenburg, Germany) is implanted via the midline approach using the facetotomy-gap. (B) This non-expandable TLIF cage consists of 2 parts connected by a hinge joint for an enlarged footprint and to be able to support the opposite side like in PLIF procedures. PLIF = posterior lumbar interbody fusion, TLIF = transforaminal lumbar interbody fusion.

Surgery was performed with patients in the prone position. After a 40 mm midline skin incision, microscopic decompression of the ipsilateral (more symptomatic) side was achieved via laminotomy. Undercutting provides ipsi- and contralateral decompression, and an obligatory contralateral facetotomy provides sufficient segmental release. Facetotomy (ipsilateral to the side of the cage to be implanted) is performed for effective decompression and to gain optimal access to the disc space for discectomy and subsequent cage implantation. After discectomy and segmental release, the reference array (Brainlab AG, Munich, Germany) was fixed to the spinous process of the distal vertebra for a 3D navigation scan. Two 8 mm skin incisions are made contralaterally, approximately 40 mm lateral to the midline (their positions are based on navigational planning and depend on the grade of the listhesis and angle of the lordosis. Therefore, the 2 contralateral pedicle screws can often be implanted through the same skin incision), and guide wires are inserted *without any anatomical landmarks* under navigated and fluoroscopic guidance in a standard posterolateral fashion (Fig. 1A). Ipsilateral K-wires were then inserted via the midline incision along a navigation-controlled trajectory from a starting point at the medial part of the junction of the superior articular process and the pars interarticularis (Fig. 1F). The more medial the entry point (ultimately depending on the length of the midline incision and the tension on the musculature), the more straightforward is the determination of the implantation trajectory via the anatomical conditions of the lamina-pedicle complex. Therefore, the ipsilateral K-wires were inserted posterior-anteriorly (vertically) following a cortical bone trajectory (CBT) (Fig. 2A). The contralateral pedicle screws and a rod were then implanted percutaneously using the Phoenix™ tower system (Orthofix, Curacao, Netherlands Antilles) followed by realignment and latching of the segment in the desired position. A polyether ether ketone (PEEK) non-expandable cage, specially designed for this procedure in 2010 (Synchro; ARCA-MEDICA, Neuenburg, Germany), was then implanted into the previously distracted intervertebral disc space through the unilateral posterior midline approach and ipsilateral facetotomy-gap (Fig. 3A–B). This non-expandable cage consists of 2 parts connected by a hinge joint for an enlarged footprint (similar to banana-shaped TLIF cages). The ipsilateral CBT screws are then inserted using the preinserted K-wires for guidance, and an ipsilaterally connecting rod was introduced to lock the realigned segmental position.

### 2.3. Measurement of the realignment and segmental ossification pattern

**Figure 4.**
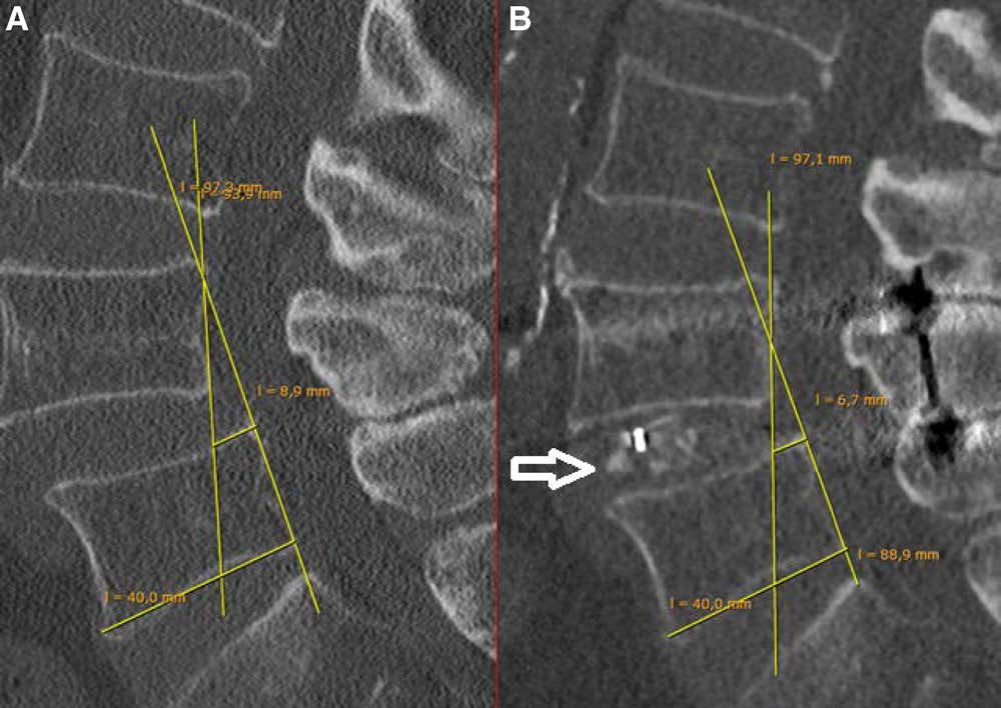
Calculation of realignment and examination of the postoperative segmental ossification (see also Fig. 5) in a patient with a Meyerding Grade I. Lines are drawn parallel to the posterior wall of the slipped vertebral body (Line 1) and the vertebral body below it (Line 2). A line (Line 3) perpendicular to Line 2 is then drawn on the upper plate which gives the slippage in millimeters. The slippage is then expressed as a percentage. (A) the preoperative slippage was 22.5%. (B) The postoperative slippage was 16.7%. The slippage improved by 24.7% (2.2 mm). The white arrow marks the interbody bony fusion at 1-year follow-up.

**Figure 5.**
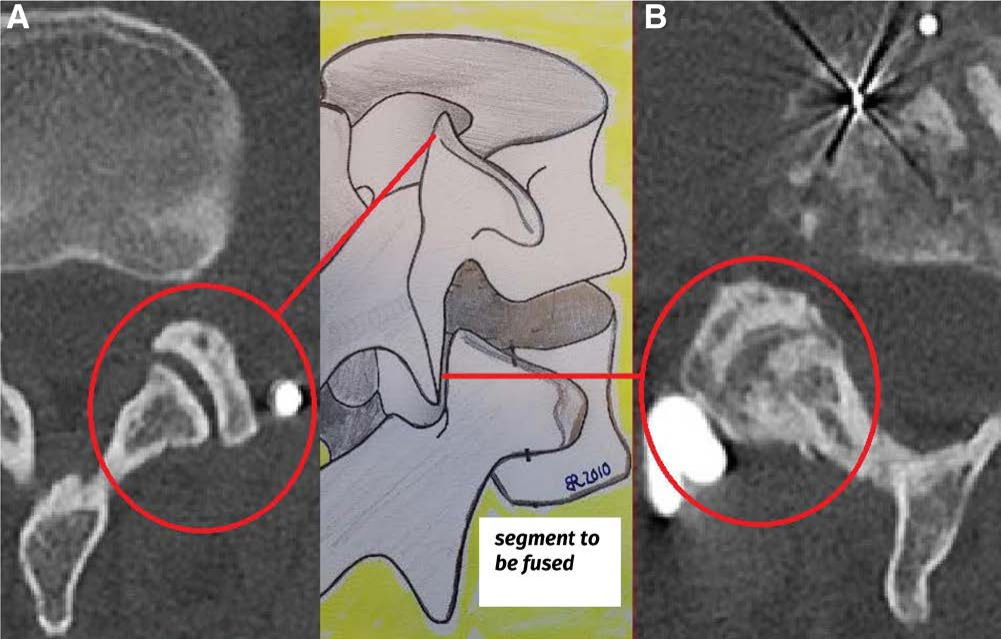
(A) Red circle: Preserved joint function at the facets located proximal to the fused segment, as it is possible to spare the joint gaps from the pedicle screws and the tulip heads with the aid of navigation. (B) Red circle: ossified joint of the fused segment. nMIS-HLIF maintains the anatomic structures of the facet joints. Opening of the facet joints and damage to the facet joint skin leads to rapid ossification of the facet joints and thus to a stabilization of the dorsal structures of the spinal column. nMIS-HLIF = navigated minimally invasive hybrid lumbar interbody fusion.

Radiological measurements of realignment were performed by comparing the pre- and postoperative anterior displacement. On a sagittal spinal computed tomography scan, a line was drawn parallel to the posterior wall of the slipped vertebra and a second line parallel to the posterior wall of the vertebra below. A 3^rd^ line was drawn perpendicular to the line on the lower vertebra and extended until it intersected the line from the slipped vertebral body. The length of the perpendicular line showed slippage in millimeters, which was then calculated as a percentage. We compared the pre- and postoperative computed tomography scans and examined the ossification patterns (Figs. 4A, B and 5B).

### 2.4. Study group

Only patients with symptomatic degenerative spondylolisthesis, Meyerding grade I to II, and unsuccessful conservative management of at least 12 months’ duration were enrolled in the study. None of the patients had previously undergone surgery at the affected level. Patients with systemic muscular disease or chronic pain syndrome were excluded. We recorded patient demographics and BMI, as well as procedure-related parameters such as surgical and procedural time, spinal level, and correction of listhesis.

### 2.5. Data acquisition and outcome measurement

All patients provided informed consent for surgery and data collection. Patient-reported outcomes (PROs) included the core outcome measures index (COMI), numerical rating scale (NRS) for the back and leg, oswestry disability index (ODI), and short form health-36 (SF-36). Follow-up was performed at 6 weeks, 3 months, 6 months, and 1 year after surgery. Furthermore, we compared the quality of life of our 1^st^ nMIS-HLIF cohort with that of the normal population (n = 6934) measured by the German Federal Statistical Office in 1998 with the SF-36 questionnaire.^[15]^ To avoid bias, the age of the control population was matched to that of our nMIS-HLIF study group (45–81 years). For statistical analysis, the values of the control population were standardized to equal zero, and preoperative deviations of the nMIS-HLIF patients from the zero points were calculated as percentages. To determine the impact of BMI on outcome measures, the study participants were grouped according to the WHO definition of obesity. There were 3 cohorts: BMI < 25 kg/m^2^, BMI 25 to 30 kg/m^2^, and BMI > 30 kg/m^2^. The values are expressed as the difference (*∆*) from the preoperative baseline.

### 2.6. Biomechanics

**Figure 6.**
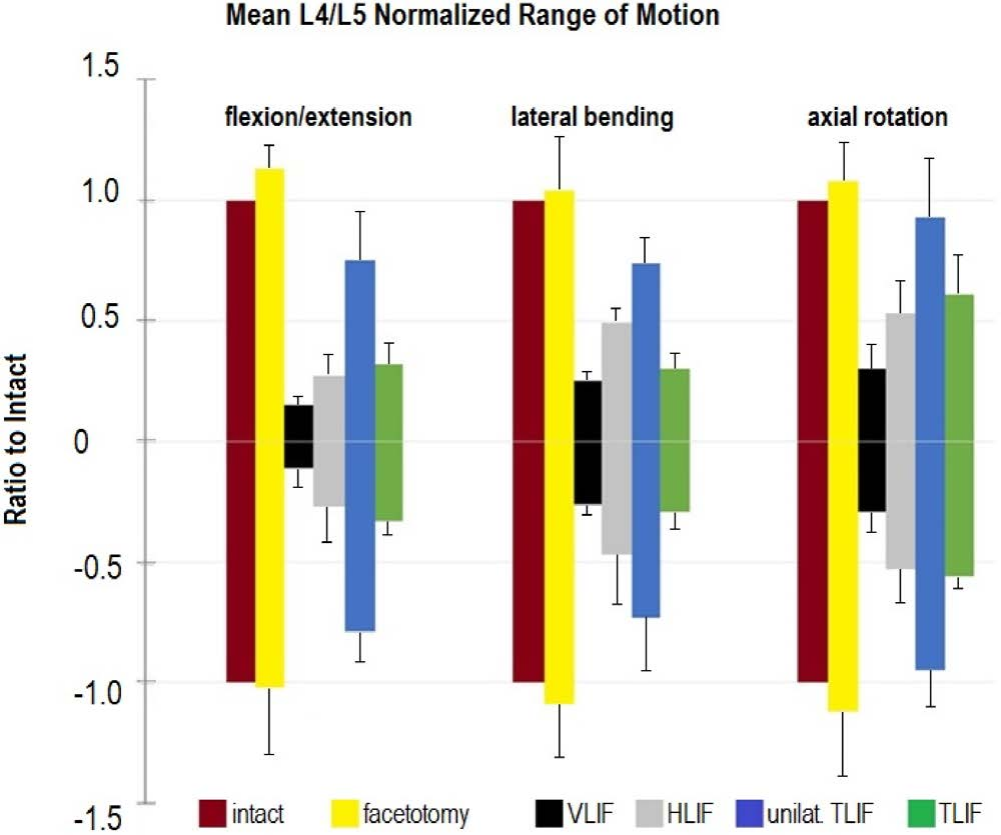
The stability of different dorsal stabilization systems (in presence of the disc and ligaments without the use of a cage) in relation to the intact and the decompressed segment was tested in *flexion/extension*, *lateral bending* and *axial rotation* and compared to each other by examination of human specimens LW4/5: intact = intact segment; facetotomy = after facetotomy of both sides and decompression; VLIF = CBT screws ipsilaterally + translaminar screw + transfacet screw (1 rode)^[15]^; HLIF = CBT screws ipsilaterally + conventional screws contralaterally (2 rods without cross-connector, because a cross-connector is usually not inserted percutaneously); unilat. TLIF = conventional pedicle screws 1 side (1 rod); TLIF = conventional pedicle screws both sides (2 rods together with angular stable cross-connector). Healthy bone conditions were examined ‐ no osteoporosis model. HLIF = hybrid lumbar interbody fusion, TLIF = transforaminal lumbar interbody fusion, VLIF = vector lumbar interbody fusion.

**Figure 7.**
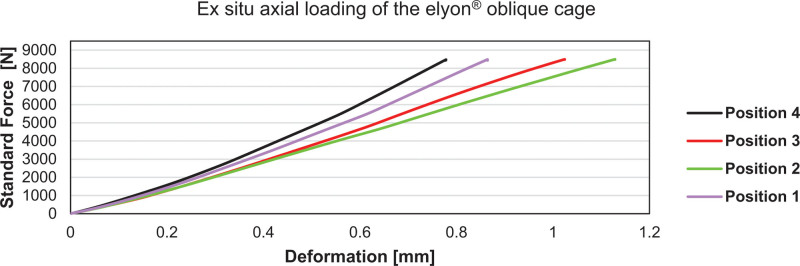
Ex situ axial loading of the elyon® oblique cage. The cage was exposed to axial loading in 4 different positions. Position 1: Cage vertically and horizontally completely expanded; Position 2: Just after the completion of the horizontal expansion and at the beginning of the vertical expansion; Position 3: Just before the completion of vertical expansion (just before reaching Position 1; Position 4: Just before the completion of the horizontal expansion. The elyon® oblique cage showed a maximal deformation < 1.2 mm with Position 2 being subject to the maximum deformation. In vertically and horizontally completely expanded form (Position 1), the deformation was slightly more than 0.8 mm. The loadbearing capacity was the highest at Position 4, just before the beginning of the vertical expansion of the cage. The experiments used material testing machine (Zwick Roell GmbH, Ulm, Germany) exerts a continuously increasing force on the materials. The machine breaks the testing after 8500 N. None of the cages showed a brittle failure (fracture).

Biomechanical tests required for the respective approval procedures on the further developed devices were conducted in accordance with the standardized test set-ups (e.g., Figs. 6 and 7).

### 2.7. Statistics

Data were analyzed using the SPSS software version 20 (IBM SPSS Statistics for Windows, Version 20, Released 2011, Armonk, NY, IBM Corp.). The analysis was performed in cooperation with the Institute of Medical Statistics at the University of Cologne, Germany. Descriptive statistics were used for the analysis of numeric variables. Independent variables were analyzed using the Mann-Whitney test and paired variables using the Wilcoxon test. Spearman’s correlation coefficients were calculated. The significance level was set at *P* < .05.

## 3. Results

### 3.1. Demographics, BMI, spinal levels, the duration of the procedure

**Figure 8.**
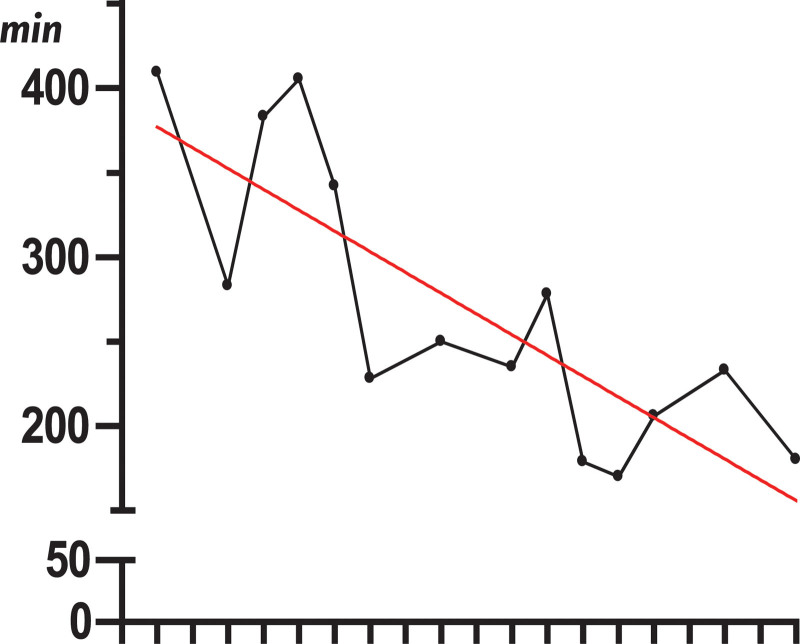
Decrease of the surgical time in the very first 14 cases.

Thirty-three patients were enrolled in this prospective study to evaluate the learning curve (Fig. 8) and the effectiveness of nMIS-HLIF. The study included 20 women (61%) with a mean age of 66.3 ± 10.5 (median 69, range 45–81) and 13 men (39%) with a mean age of 66.4 ± 10.5 (median 69, range 45–78). There was no significant age difference between the sexes (*P* = .89).

Twenty-eight patients (85%) completed the entire follow-up period. Five patients (15%) were lost to follow-up. We defined patients who had not completed any questionnaires at 6 months or 1 year as “lost to follow-up.” Two of these patients (6%) withdrew their consent, 1 (3%) had developed dementia, 1 (3%) underwent further spinal surgery at another hospital, and 1 (3%) was diagnosed with a mitochondrial disease.

The mean BMI was 28.2 kg/m2 ± 5.53 (median 27.6, range 18.9–40.6). Twenty-three (70%) patients were either pre-obese or had grade I to III obesity according to the WHO classification.

L4/5 and L5/S1 were the most commonly affected segments (83%), with 58% of operations performed at L4/5. The mean duration of the whole procedure was 239 ± 69.8 (median 233, range 150–405) minutes. There was a significant negative correlation (*P* = .011) between the duration and chronological order of the procedures, suggesting a successful learning curve for the surgical team.

### 3.2. Reduction of the listhesis and ossification pattern

The mean listhesis given as percentage was 22.0 ± 6.7 (median 21.5%, range 11.1–36.1) preoperatively and 13.6 ± 13.62 (median 15.2%, range 0.0–27.2) after correction. This reduction of 8.4% was statistically significant (*P* < .01). A significant correlation was observed between the reduction and NRS Leg (*P* = .004) and the SF-36 Physical Component Summary (*P* = .016), but not in the other PRO parameters at follow-up (*P* > .05). In all 28 patients, ossification of the remaining facet joints was observed (Fig. 5B), while a clear bony fusion (Fig. 4B) of the disc space was not achieved after 1 year in 9 patients. There was no correlation between patient-specific ossification pattern and outcome.

### 3.3. Patient reported outcomes (PROs)

**Figure 9.**
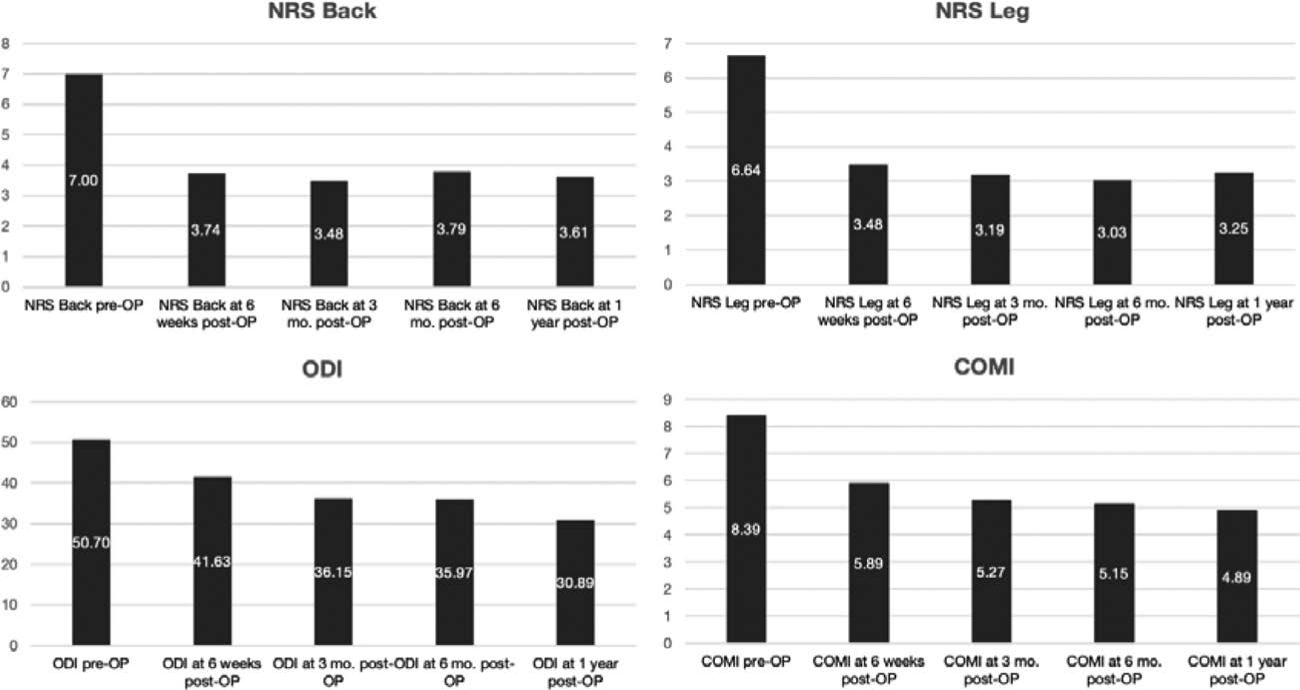
Patient Reported Outcomes (PROs) showed an improvement in NRS Back, NRS Leg, ODI and COMI. The NRS Back (*P* < .001) and NRS Leg (*P* < .001) improved by approximately 50% at all times. ODI und COMI declined throughout the follow-up period; the difference from baseline was highly significant at the 1-year follow-up (*P* < .001 in each case). COMI = core outcome measure index, NRS = numeric rating scale, ODI = Oswestry disability index.

Overall, significant postoperative improvement was observed in all PROs examined (Fig. 9). The mean preoperative NRS Back score was significantly improved at all times (*P* < .001), decreasing from 7.0 ± 2.3 (median 8, range 0–10) before surgery to 3.6 ± 3.0 (median 3.5, range 0–9) after 1 year. A similar pattern was observed for the mean NRS Leg score, which was also significantly improved at all times (*P* < .001), decreasing from 6.6 ± 2.9 (median 8, range 0–10) preoperatively to 3.3 ± 2.8 (median 3, range 0–9) at the 1-year follow-up.

Compared with the preoperative scores, both the mean ODI and the mean COMI displayed a continuous decline throughout the follow-up period, and this was also significant at all times (*P* < .001). The mean ODI fell from 50.7 ± 14.9 points (median 49, range 20–78) preoperatively to 30.9 ± 17.0 (median 27, range 0–62) at the 1-year follow-up, showing an improvement of approximately 39%. The mean COMI decreased from 8.39 ± 1.34 points (median 8.4, range 5.56–10) preoperatively to 4.89 ± 3.26 (median 4.1, range 0–9.8) showing an improvement of approximately 42% over the same time.

**Figure 10.**
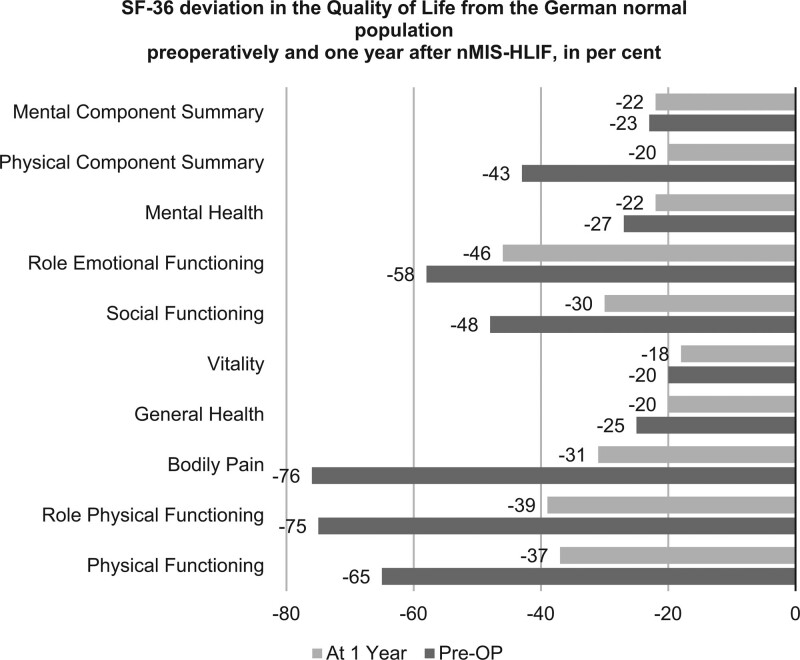
Quality of Life and Health as measured by the SF-36 questionnaire. Preoperatively the items Bodily pain (-76%), Role functioning/physical (-71%) and Physical functioning (-65%) affected daily life the most. Their amplitude declined significantly to -31% for Bodily pain (*P* < .001), -39% for Role functioning/ physical (*P* = .003), and to -37% for Physical functioning (*P* = .002). Pain reduction by 45% was the most marked. The Physical component summary improved from 44% to 19% (*P* < .001). Social functioning improved from -48% to—30% (*P* < .015). Other important parameters such as General health, Mental health, Role functioning/emotional, and Vitality also improved (*P* > .05). SF-36 = Short form health 36.

The baseline values of the nMIS-HLIF study group deviated from the normal population in all aspects of the SF-36 questionnaire (Fig. 10). The greatest differences were observed in the parameters “Bodily pain” (76%), “Role functioning/physical” (71%) and “Physical functioning” (65%). The amplitude of all these deviations had undergone a significant reduction at the 1-year follow-up. The deviation in “Bodily pain” sank to 31% (*P* < .001), “Role functioning/physical” to 39% (*P* = .003) and “Physical functioning” to 37% (*P* = .002). At the 1-year follow-up, the difference in the physical component summary” improved from 44% to 19% (*P* < .001). Social functioning also improved substantially after 1 year (*P* < .015). The greatest reduction in deviation from the normal population was seen in the parameter “Bodily pain,” which improved by 45%. The parameters “General health,” “Role functioning/emotional,” “Mental health” and “Vitality” all improved, albeit without reaching statistical significance. Overall, the patients reported an impressive improvement in their quality of life.

### 3.4. Relation between outcome measures and the BMI

In all 3 BMI cohorts, PROs improved at the 1-year follow-up. This improvement was more pronounced in patients with a normal weight (BMI < 25 kg/m^2^) than in those with a BMI > 30 kg/m^2^. This difference between BMI < 25 kg/m^2^ and BMI > 30 kg/m^2^ was significant in COMI (*P* = .023), NRS leg (*P* = .003), and ODI (*P* = .036). Pre-obese patients (BMI 23 kg/m^2^–30 kg/m^2^) also had better outcomes than obese patients (*P* = .018).

### 3.5. Complications

The complication rate was 15.2% (n = 5). Durotomy (9.1%, n = 3) was the most common complication. One percutaneously inserted pedicle screw malposition with revision surgery and 1 wound infection without revision were found (Table 1).

**Table 1:** Complications occurred in 5 patients (15.2%). Durotomy was the most frequent (n = 3, 9.1%). Pedicle screw malposition occurred in 1 patient and a wound infection in another.

Complications	n	%
Pedicle screw loosening in long term	0	0,0%
Pedicle screw malposition with revision surgery	1	3,0%
Durotomy	3	9.1%
Cage malposition with revision surgery	0	0.0%
Permanent radiculitis	0	0.0%
**Total**	**5**	**15.2%**

### 3.6. Further developments

**Figure 11.**
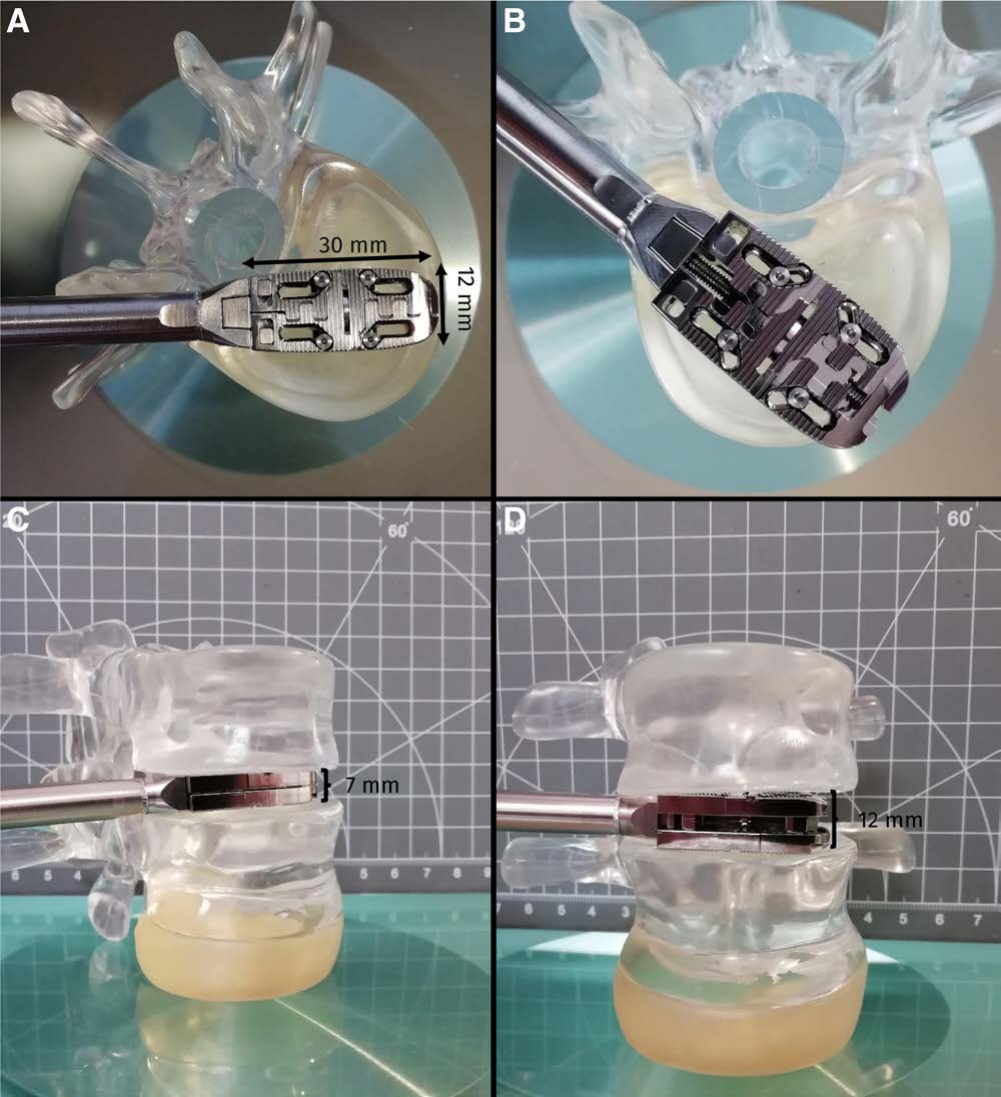
Elyon® oblique cage developed by our Prospective Spine® working group to support minimally invasive stabilization procedures like nMIS-HLIF and to avoid accidental durotomy. (A) In the insertion mode, the cage has a minimum height of 7 mm and a width of 12 mm. After its implantation into the intervertebral disc space, the cage almost doubles its surface area (B) for a larger footprint and it can then be raised continuously (C, D) for a good press fit and better sagittal balance, without the need for any sample cages. It might reduce the frequency of durotomies and protect the medial ipsilateral facet in nMIS-HLIF and nMIS-VLIF. nMIS-HLIF = navigated minimally invasive hybrid lumbar interbody fusion, nMIS-VLIF = navigated minimally invasive vector lumbar interbody fusion.

The surgical consideration of the modified surgical procedure with biomechanically improved cortical screw anchoring (Fig. 2A) and the complication spectrum (especially durotomies) led to the development of the (navigation guided minimally invasive vector lumbar interbody fusion: Fig. 2B, C) and to a family of expandable intervertebral body implants (Figs. 2D, 7, and 11).^[16]^

## 4. Discussion

Different surgical approaches have been developed to achieve stabilization in degenerative listhesis to improve sagittal alignment and provide better functional outcomes. Minimally invasive procedures have the advantage of conserving the musculature and associated vascular and neural structures, but depend on percutaneous insertion of pedicle screws without any anatomical landmarks. A minimally invasive posterior stabilization procedure that can be performed from only 1 side protects the muscles of the opposite side, but also allows effective minimally invasive contralateral decompression. At the same time, introducing spinal navigation supports both percutaneous insertion of conventional pedicle screws and open implantation of unfamiliar CBT screws.

Reinshagen et al reported this procedure in 2015 and called it a minimally invasive hybrid lumbar spinal fusion technique, where the term hybrid refers to the surgical approach, because, as in PLIF, the minimally invasive approach via the midline facilitates bilateral decompression while preserving the anatomical integrity of both facet joints and the specially designed cage supports both sides.^[13]^ On the other hand, the approach is only from 1 side in order to protect the muscles on the opposite side, as in TLIF. However, the use of 2 different screw directions within the same surgical procedure is a combination, rather than hybridization.

The present study was designed to verify the learning curve of the surgical team (Fig. 7) and the effectiveness of the surgical modification as a first step towards a single-port endoscopic lumbar stabilization method.

The conventional screw direction of pedicle screws, as described by Roy Camille in the 1970s, is in the dorsolateral or coaxial direction.^[17,18]^ He discovered the pedicle as an anchorage site for screws with the realization that the pedicle is the strongest part of the vertebra.

This common screw direction tends toward the recess, which houses the passing nerve root of the segment to be fused, so that recess-related screw positions occur in both percutaneous and open procedures (Table 1).

Conventional pedicle screw anchors only once in the cortical bone, and the longer lever of the screw is surrounded by cancellous bone. Why not use longer cortical structures, especially in the presence of osteoporosis, and guide the screws along the cortical bone trajectory (CBT) with the aid of navigation (Fig. 2A)?^[16]^ In addition, CBT screws angle away from the recesses, which reduces the risk of paresis due to a recess-related insertion of pedicle screws. In case of any destruction of the pedicular structure by metastatic processes, other cortical structures can also be included in the stabilization (Fig. 2B). If all screws are cortically multi-anchored, and 2 CBT screws are ipsilaterally connected together with 1 translaminar and 1 transfacet screw via a single port for single-level fusion (Fig. 2C), a biomechanically superior stabilization system is achieved (Fig. 2.1), which enables single-port endoscopically assisted lumbar fusion systems.

### 4.1. Assessment of the procedure with regard to the learning curve

Percutaneous insertion of pedicle screws is technically more difficult and requires more experience than their placement in open procedures because of the visual lack of anatomical landmarks. With nMIS-HLIF for single-level stabilization, the surgeon performed percutaneous, navigation-guided implantation of the 2 contralateral common pedicle screws and an open insertion of the 2 ipsilateral cortical bone trajectory screws under navigational control. Therefore, nMIS-HLIF is an ideal procedure for surgeons who want to train a percutaneous minimally invasive stabilization procedure. Within the same procedure, the surgeon can perform percutaneous pedicle screw implantation and open CBT-screw insertion under navigation control, which trained surgical perception and led to a quick reduction in surgical time (Fig. 7).^[19]^

### 4.2. Outcome

In this study, we have reported on the PROs and surgical results of the first 33 patients who underwent nMIS-HLIF. PROs revealed a significant reduction in pain and other important quality-of-life parameters.

With a range of commonly used back pain assessment tools (including the ODI and NRS), Ostelo et al proposed that at least a 30% change from the baseline should be achieved for improvement to be considered clinically meaningful.^[20]^ Our study found 48.6% improvement in NRS Back, 50% in NRS Leg, 39.1% in ODI, and 41.7% in COMI. These patient-reported outcomes confirm that nMIS-HLIF meets the minimum requirements for meaningful improvement and patient comfort. Nevertheless, our improvement rates were lower than those described in the literature. Wong et al compared MIS-TLIF (n = 144, mean age 61) with open TLIF (n = 54, mean age 58) over a mean follow-up period of 45 months; in the MIS-TLIF cohort, VAS back, VAS leg, and ODI improved by 83%, 87%, and 66%, respectively.^[21]^ in the open TLIF cohort, the corresponding improvements in VAS back, VAS leg, and ODI were 75%, 85%, and 59%, respectively. This study did not take the BMI of the cohort into account. The authors also stated that the same team had already operated on 100 other patients in the initial phase of the study between 2002 and 2004. This information suggests that their learning curve was complete, while in the current study, the learning curve was at an earlier stage. Taking the 33 cases of the present study as the first cases necessary for the learning curve of the surgical team, a better outcome was also recorded after nMIS-HLIF in further 132 patients operated on over the next few years.^[19]^ In yet another study comparing MIS-TLIF (n = 57) with open TLIF (n = 11), the MIS-TLIF patients had a 30.9% improvement in ODI while the open TLIF patients had an 11.7% improvement at the 1-year follow-up.^[22]^ The figures for improvement in VAS were 56.2% and 30.1% respectively. The MIS-TLIF cohort had a mean age of 61.1 years and a mean BMI of 30, while the open TLIF group had a mean age of 56.4 years and a mean BMI of 30. These results are comparable to ours and underline the importance of conserving midline structures and musculature as much as possible to allow postoperative functional recovery. Using a PLIF procedure, Yang et al reported a significant reduction of 67% in ODI at the final assessment (mean follow-up time 30.5 months) and a reduction of 74% in VAS in their series of patients (n = 34, mean age 42.7 years) with isthmic spondylolisthesis (L4/5 or L5/S1) undergoing an open PLIF procedure (single-level fusion).^[23]^ Wegmann et al reported a 57% improvement in the COMI at the 12-months follow-up and 42% improvement after 24 months in their PLIF series (n = 43, mean age 55.5 years, single-level fusion L4/5 or L5/S1, degenerative spondylolisthesis).^[24]^ Regarding ODI, Wegmann et al reported a 57% improvement at 12 months and a 42% improvement at 24 months, while the SF-36 physical component summary improved by 29% at 12 months and by 27% at 24 months.^[24]^ Neither study reported the BMI in their cohorts. In their series of patients with lumbar degenerative spondylolisthesis undergoing PLIF procedure (n = 44, mean age 58.35, BMI 25–29.9 75%, BMI > 30 4.5%), Farrokhi et al reported a 53% improvement in ODI at the 12-month follow-up and a 66% improvement at 24 months.^[25]^ Overall, the improvement in terms of PROs in our initial nMIS-HLIF study group was somewhat less pronounced than in the abovementioned recent studies and the patients in this study were substantially older than those in the cited studies.

### 4.3. Biomechanics

The MIS-HLIF uses a tripod, consisting of a fused intervertebral disc space and 2 ossified facet joints, for the static design of stabilization, which may be particularly useful for older patients with manifest osteoporosis. Cortical anchoring of the ipsilateral pedicle screws also contributed to improved statics (Fig. 2.1). In 10 years of clinical use of MIS-HLIF in degenerative listhesis, there was no loosening of the internal stabilization system. Pedicle screws were only loosened in cases of genuine listhesis (these patients were not participating in the present study) with an interruption of the bony vertebral arch. This complementary observation may support the thesis of any static benefit of the preserved posterior bony structures for stabilization, because in patients with genuine listhesis, the bone substance is better due to the considerably younger average age.

### 4.4. Obesity

Obesity is an ever-growing public health concern that directly affects the decisions and practices of spinal surgeons. However, the extent to which BMI and obesity impact the perioperative and long-term outcomes of patients is still a matter of debate. Recent literature reports controversial results. While some authors^[26–28]^ did not find that obesity or morbid obesity had a substantial impact on long-term outcomes, others^[29,30]^ did observe a relevant negative impact. Common to all these studies is the higher incidence of postoperative complications in obese patients. Minimally invasive and open fusion procedures achieved similar results in terms of outcome in patients with BMI > 25 kg/m^2^.^[11,31,32]^ However, minimally invasive procedures had markedly fewer complications in these studies. The subgroup analysis of the SPORT study (a retrospective registry study with 601 patients) concluded that the duration of the operation, wound infections, and reoperation rates were significantly higher in patients with a BMI > 30 kg/m^2^.^[33]^ But the outcome at the 4-year follow-up was not significantly worse in these patients. The surgical time in our study was also significantly longer in patients with a BMI > 30 kg/m^2^ (*P* = .016). Furthermore, a recently published study comparing the outcomes of obese (n = 382) and non-obese (n = 415) patients undergoing surgery for grade I lumbar spondylolisthesis concluded that obesity is associated with worse NRS leg pain and quality of life.^[34]^ However, obese patients reported significantly better PRO results at the 12-months follow-up.

In agreement with this subgroup analysis and the recent data, the baseline preoperative PRO scores (SF-36, ODI, and NRS Back) in our study group were higher than those of patients with normal weight. The improvement in the SF-36 parameter “Physical functioning” was significantly lower in patients with a BMI > 30 kg/m2 than in those with normal weight (*P* = .03). Furthermore, our subgroup analysis (BMI < 25 kg/m^2^ vs BMI 25–30 kg/m^2^ vs BMI > 30 kg/m^2^) revealed a significant correlation between BMI and PROs (COMI *P* = .008, NRS Leg *P* = .009, SF-36 “Pain” *P* < .001 and “Physical component summary,” *P* = .003). Patients with a BMI < 25 kg/m^2^ and BMI 25-30 kg/m^2^ reported substantial improvement from baseline. Nevertheless, patients with a BMI > 30 kg/m^2^ experienced relief of their symptoms, although in terms of PROs, it was not as pronounced as in patients with a BMI < 25 kg/m^2^. BMI was not associated with postoperative complications in our study group (BMI < 30 kg/m^2^ vs BMI > 30 kg/m^2^, *P* = .66).

#### 4.4.1. *Reduction of the listhesis*.

There is still some controversy regarding the impact of listhesis correction on patient outcomes. While Wegmann et al observed a correlation between a favorable outcome and the extent of listhesis reduction from 34.2% to 15.1% at the 1-year follow-up in a cohort of 40 patients undergoing PLIF, Benli et al did not find any such correlation in their prospective randomized study of 40 patients undergoing posterolateral decompression and instrumented fusion (without cage implantation; 20 patients with low-grade spondylolisthesis and 20 with high-grade spondylolisthesis).^[24,35]^ Similarly, Hagenmaier et al also reported that listhesis reduction did not lead to a better outcome in their cohort of patients with low-grade spondylolisthesis (n = 36 Meyerding I and n = 36 Meyerding II) undergoing posterolateral decompression and fusion with instrumentation (but no cage implantation).^[36]^ Furthermore, a recent study by Balasubramanian et al also did not observe a positive effect of reduction grade on the outcome in patients undergoing TLIF.^[37]^

In our patients, we observed a reduction from 22.0% to 13.6%, similar to the results in the current literature. We found a significant correlation between the reduction and NRS Leg (*P* = .004) and the SF-36 Physical Component Summary (*P* = .016). However, the reduction rates on COMI, ODI, NRS back, and the remaining SF-36 showed no homogenous correlations. BMI was a significant intrinsic factor influencing the degree of listhesis reduction. A higher BMI was significantly associated with a lower degree of reduction (*P* = .032). The higher the BMI, the longer the surgical access route and the more unfavorable the angle ratios for the undercutting maneuver for a given size of surgical incision. The more unfavorable these angle ratios, the more difficult the minimally invasive facetotomy of the opposite facet, which may lead to an incomplete segmental release. Poorer release in obese patients resulted in less reduction with the given instruments.

Our literature search revealed only 1 study, albeit with an anterior lumbar interbody fusion procedure, which found a significant immediate positive and short-term effect of BMI < 18 (3.8 mm) versus BMI > 25 (2 mm) (*P* = .004) on the degree of reduction, with a BMI < 18 being defined as underweight.^[38]^

### 4.5. *Complications*

A meta-analysis by Joseph et al with 5454 patients, found the incidence of complications after MIS-TLIF to be 19.2%.^[9]^ In their meta-analysis, Tian et al examined the outcomes of 785 patients who underwent MIS-TLIF or oTLIF and reported an incidence of 17.6% and 16.8%, respectively.^[39]^ In their retrospective study of 513 patients, Wong et al reported an incidence of 15.6% after minimally invasive lumbar fusion procedures.^[40]^ In our study, we found a complication incidence of 15.2%, with durotomy (9.1%) being the most common complication. Incidental durotomy is reported to be between 2.27% and 5.1% in the current literature.^[18,39,40]^ Another meta-analysis found a higher durotomy rate in oPLIF than in oTLIF (*P* = .01).^[41]^ Since nMIS-HLIF uses some aspects of PLIF, it tends to have a comparable complication profile. The complication rate depends on what is defined as a complication; Wang et al reported a perioperative complication rate of 36,76% in 204 MIS-TLIF procedures.^[42]^ A possible explanation for our higher incidence of dural tears is that the unilateral posterior midline approach of nMIS-HLIF only removes part of the ipsilateral facet joint, so that the sample cages and definitive cage are inserted in direct proximity with the dura. If cage heights significantly > 7 to 8 millimeters are required to improve sagittal balance and reduce listhesis, cage implantation may lead to durotomy. In our study group, there was only 1 (3%) superficial wound infection, which healed without any additional surgical intervention, confirming earlier studies.^[35]^ This 1 patient had a BMI of 40 kg/m^2^ and a surgery time longer than the mean. Wound infections can be differentiated into superficial and deep infections, with the latter usually requiring surgical intervention. Wong et al reported 4.2% superficial and 0% deep wound infections for MIS-TLIF, with 12.9% and 5.6%, respectively, for open TLIF.^[21]^ In their series of 62 patients and single-level surgery (n = 30 MIS-TLIF; n = 32 oTLIF), Shunwu et al reported a superficial infection rate of 9.4% and a deep wound infection rate of 0% for MIS-TLIF versus 3.3% and 3.3%, respectively, for oTLIF.^[43]^ The low wound infection rates of minimally invasive procedures, including nMIS-HLIF, may be explained by the smaller skin incisions of these procedures. The malposition of the pedicle screw was a recess-related insertion of a conventional pedicle screw that had to be revised because of postoperative pain due to constriction of the nerve root in the recess. Using a more cortical bone trajectory for screw insertion may reduce the risk of harassment or injury to the nerve root.

### 4.6. Duration of the procedure

Surgical time is widely regarded as a measure of the steepness of the learning curve and varies in the literature. For single-level MIS-TLIF procedures, mean surgical times of 123 minutes, 166 minutes, and up to 204 minutes have been reported.^[18,44,45]^ The duration of the operation tends to be longer for open procedures.^[44]^ The mean skin-to-skin time of 239 ± 69.8 minutes refers to the time for the whole procedure including the 3D scan, data transfer to the navigation unit, and performance of the navigation. Optical navigation proved to be very susceptible to disturbances, so that more than a 3^rd^ of the procedural time had to be spent on the navigation. Over time, the operation duration decreased to 155 to 182 min. (standard deviation  ± 27 min) In a further series of 132 patients operated on with the nMIS-HLIF technique using the same equipment.^[19]^ We did not perform a laminectomy, but a more elaborate decompression of the segment to preserve both facets. The special segmental decompression in nMIS-HLIF with an ipsilateral facetotomy and opening of the contralateral facet during the undercutting maneuver means that the bony structures of both facets and the vertebral arch are preserved. The segment stabilizes faster by rapid ossification of the facets than was the case when the segment had to be stabilized only by ossification of the disc space after laminectomy (Fig. 5B), on the other hand, the facet joints of the adjacent not to be fusioned segment are preserved, since the tulip head of the pedicle screws and the rods can be placed without putting stress on the adjacent level facet joints (Fig. 5A).^[19]^

After the ossification of the remaining facet joints, there is a natural fixation of the elastic module of the bone (ossified posterior column), and the internal fixation device made of titanium can subsequently be removed in a small surgical procedure to protect the adjacent segments. Dividing the nMIS-HLIF procedures into an early and late group, as suggested by Lee et al, the surgical time fell from 256 ± 69.3 minutes to 223 ± 69.3 minutes.^[45]^ However, this reduction was not significant (*P* = .15), but in the first half of the 28 patients, a relatively rapid decrease in the surgery time was observed (Fig. 7). Lee et al, in their series with twice the number of patients that we had, achieved a reduction from 254 ± 44 minutes to 183 ± 23 minutes.^[44]^ Considering the time for the procedure in terms of the affected spinal segments and PROs, there was no significant difference. In addition, the allocation into the early or late group revealed no significant difference with regard to outcome. These findings are consistent with those of the current literature. At the beginning of the changeover from the open procedure to the nMIS-HLIF, approximately 1-3^rd^ of the procedural time is needed for the intraoperative 3D scan and the adjustment of the optical navigation, which is susceptible to interference. This time is well invested in view of the comparable outcomes, the surgeons’ learning curve, and minor radiation risk for the surgical team. In addition, magnetic-induction-based navigation and spinal robotics will offer improved and faster spinal navigation in the future.

### 4.7. Study group

The mean age of our patient cohort was 66.3 years and hence 6 years older than patients in an American degenerative spondylolisthesis registry with approximately 50,000 operations.^[46]^ The sex distribution was equivalent to that of the registries in the USA and Denmark.^[1,46]^ The distribution of the affected spinal levels with L4/5 (58%) occurring frequently in our cohort was also comparable to the literature.^[45]^ The mean BMI of 28.2 ± 5.5 kg/m^2^ in our cohort was, however, > the BMI of the Danish registry (24.4–26.6 kg/m^2^).

### 4.8. Ongoing developments

To further improve the procedure and avoid complications, especially dural tears, our Prospective Spine^®^ group has developed a proprietary expandable oblique cage (elyon®) with an initial height of 7 mm, an initial width of 12 mm, and a length of 30 mm (Fig. 10A, 10C). After implantation, the cage width doubles in size, and the cage can then be expanded in height to reach an optimal pressfit (Fig. 10B, 10D). A recent ex vivo test demonstrated the cage’s favorable axial load-bearing characteristics throughout its different expansion settings (Fig. 6). In addition to decreasing the risk of dural tear during implantation due to its expandable nature, we regard the development of suitable expandable devices as a prerequisite for the introduction of endoscopically assisted single-port stabilization systems.

### 4.9. Limitations of the study

These are the results of the first 28 (5 lost to follow-up) patients who underwent surgery using the nMIS-HLIF technique. Long-term follow-up data (> 2 years) were not within the scope of this study.

The study was performed during the learning curve of 1 neurosurgeon. Surgical skills and familiarity with the technique would affect the outcomes.

## 5. Conclusions

This study describes the first 33 patients and surgical outcomes after nMIS-HLIF, a modified minimally invasive, navigated posterior spinal approach, which combines the advantages of TLIF and PLIF procedures. Significant improvements in NRS back, NRS leg, COMI, ODI, and SF-36 physical functioning and a reduction of listhesis by 8.4% were observed. The steepness of the learning curve and complication rate were comparable to those of other minimally invasive procedures. The complication spectrum and specific surgical requirements of the nMIS-HLIF procedure facilitated the development of an expandable cage, which demonstrated favorable ex vivo axial load-bearing characteristics. The nMIS-HLIF procedures and the experiences gained during its development have been essential in our ongoing efforts to develop an endoscopically assisted single-port lumbar fusion system.

## Author contributions

**Conceptualization:** Bernhard Rieger.

**Data curation:** Agrin Esmael.

**Formal analysis:** Kerim Hakan Sitoci-Ficici.

**Investigation:** Uta Brautferger, Radovan Hudak, Jozef Zivcak.

**Methodology:** Kerim Hakan Sitoci-Ficici, Daniel Ruess, Marek Molcanyi, Radovan Hudak, Jozef Zivcak, Bernhard Rieger.

**Supervision:** Clemens Reinshagen, Gabriele Schackert, Bernhard Rieger.

**Validation:** Thomas Pinzer.

**Visualization:** Uta Brautferger, Marek Molcanyi.

**Writing – original draft:** Kerim Hakan Sitoci-Ficici.

**Writing – review & editing:** Kerim Hakan Sitoci-Ficici, Hongzen Jiang, Clemens Reinshagen, Bernhard Rieger.
